# The SUSTech-SYSU dataset for automated exudate detection and diabetic retinopathy grading

**DOI:** 10.1038/s41597-020-00755-0

**Published:** 2020-11-20

**Authors:** Li Lin, Meng Li, Yijin Huang, Pujin Cheng, Honghui Xia, Kai Wang, Jin Yuan, Xiaoying Tang

**Affiliations:** 1 Department of Electrical and Electronic Engineering, Southern University of Science and Technology, Shenzhen, 5180000 China; 2 State Key Laboratory of Ophthalmology, Zhongshan Ophthalmic Centre, Sun Yat-sen University, Guangzhou, 510000 China; 3 School of Electronics and Information Technology, Sun Yat-sen University, Guangzhou, 510000 China; 4 Department of Ophthalmology, Gaoyao People’s Hospital, Zhaoqing, 526000 China

**Keywords:** Retinal diseases, Diagnosis, Machine learning

## Abstract

Automated detection of exudates from fundus images plays an important role in diabetic retinopathy (DR) screening and evaluation, for which supervised or semi-supervised learning methods are typically preferred. However, a potential limitation of supervised and semi-supervised learning based detection algorithms is that they depend substantially on the sample size of training data and the quality of annotations, which is the fundamental motivation of this work. In this study, we construct a dataset containing 1219 fundus images (from DR patients and healthy controls) with annotations of exudate lesions. In addition to exudate annotations, we also provide four additional labels for each image: left-versus-right eye label, DR grade (severity scale) from three different grading protocols, the bounding box of the optic disc (OD), and fovea location. This dataset provides a great opportunity to analyze the accuracy and reliability of different exudate detection, OD detection, fovea localization, and DR classification algorithms. Moreover, it will facilitate the development of such algorithms in the realm of supervised and semi-supervised learning.

## Abstract (summary)

**Table:** 

Measurement(s)	diabetic retinopathy
Technology Type(s)	machine learning
Sample Characteristic - Organism	Homo sapiens
Sample Characteristic - Environment	hospital • laboratory environment
Sample Characteristic - Location	Guangdong Province

Machine-accessible metadata file describing the reported data: 10.6084/m9.figshare.13168106

## Background & Summary

Diabetic retinopathy (DR) is one of the microvascular complications of diabetes mellitus and a leading cause of blindness among working-age adults in developed countries^1^. It is estimated that currently 463 million adults in the age range of 20–79 years have diabetes, and this number will reach 700.2 million by 2045^2,3^.

**Fig. 1:**
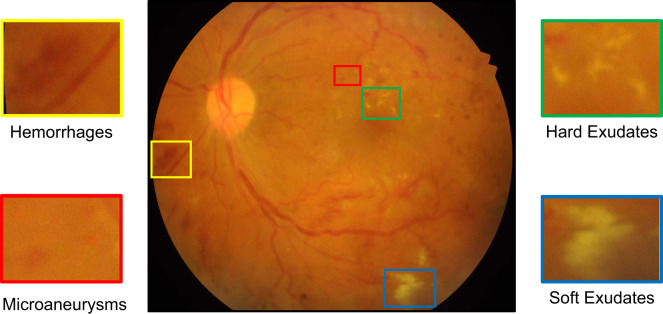
A representative fundus image with the four most common types of DR lesions: Hemorrhages, Microaneurysms, Hard Exudates, and Soft Exudates.

DR lesions include microaneurysms, hard exudates, soft exudates, hemorrhages, intraretinal microvascular abnormalities, neovascularization and so on, the most common ones of which are shown in Fig. 1. Hard exudates and soft exudates^4–6^ typically manifest in an early stage of DR. Hard exudates are mainly composed of extracellular lipid, and are usually located in the outer layer of the retina. They can be either individual dots, continuous flaky spots, or circumferential lesions surrounding retinal edema or microaneurysm. Soft exudates are localized edema or infarcts in the nerve fiber layer. In fundus images, they appear white or pale yellow, having a round or elliptic shape, with fuzzy edges. Research has demonstrated that the area and amount of hard exudates can serve as potential discriminant indicators of the severity of DR^7^. And an increase in the number of hard exudates has been suggested to be associated with an increased risk of vision loss^8,9^ as well as subretinal fibrosis in diabetic macular edema (DME)^10^.

In DR, an early detection and timely intervention is vital for protecting a patient’s visual function. Recent technological advancements in big data, computing power, and machine learning technologies have enabled fast and efficient computer-aided diagnoses of DR, wherein identification and quantization of exudates are essential components. During the past decade, various methods, which can be roughly divided into four categories (thresholding methods^11^, region growing methods^12^, morphology methods^13^, and machine learning methods^14^), have been developed for automatically detecting exudates. Machine learning methods, especially those with deep convolutional neural network architectures, have achieved overwhelming performance. Machine learning methods depend considerably on the sample size of training data and labels’ quality. Therefore, creating high-quality and large-scale training data has become a significant research direction in ophthalmic image analysis. For instance, the *ORIGA*^−*light*^ dataset was constructed for optic disc and optic cup segmentation^15,16^. DRIVE and STARE are two classic fundus datasets for retinal vessel segmentation, and STARE also provides diagnostic information for a larger set of fundus images^17–20^. In one of our previous works, we also developed a dataset containing 712 ocular staining images for corneal ulcer segmentation and classification^21,22^. However, to the best of our knowledge, existing large-scale and well-annotated fundus image datasets with lesion annotations are relatively limited.

Segmentation and detection are the two most popular approaches for lesion identification. There are several differences between them: (1) Segmentation methods require pixel-level annotations, while the latter requires bounding boxes or contours; (2) Segmentation methods often require more computing resources and training-testing time; (3) The outputs of segmentation methods are often more precise. Although pixel-wise annotations have a higher labeling accuracy, the bounding or contouring approach for detection is more practically feasible and efficient. Clinically, both segmenting and detecting lesions are beneficial to quantify the severity of DR. Currently, there are several publicly-available datasets for exudate identification. The DIARETDB1_v2 dataset contains 46 fundus images with rough polygonal boundary annotations for exudates^23,24^. HEI-MED^25,26^, consisted of 169 samples, is constructed for detecting exudates in DME. They share common problems: the annotations are not precise enough for a segmentation purpose, and the sample sizes are relatively limited for training detection models (one fundus image is usually treated as one sample). The e-Ophtha EX and IDRiD datasets have more precise annotations on exudates at a pixel-level, but they are composed of only 47 and 81 fundus images^27–30^.

In such a context, we develop a large-scale DR dataset, containing fundus images and the corresponding exudate detection annotations, left-versus-right eye labels, DR grades, the bounding boxes of OD, and fovea locations. This dataset will provide an excellent opportunity for developing and validating automated exudate detection algorithms, as well as DR classification algorithms. Furthermore, it can also be used for designing and testing OD identification and fovea localization pipelines. Overall, the dataset we construct in this paper provides a powerful resource for anatomical landmark detection, lesion detection, and DR classification based on fundus images.

## Methods

### Data collection

A total of 603 fundus images from DR patients and 631 fundus images from healthy people were collected from the Department of Ophthalmology, Gaoyao People’s Hospital and Zhongshan Ophthalmic Center, Sun Yat-sen University. All participants provided written informed consent complying with the approval requirements of the Medical Ethics Committee at Gaoyao People’s Hospital and Zhongshan Ophthalmic Center. This study followed the tenets of the Helsinki Declaration and was approved by the Medical Ethics Committee, Gaoyao People’s Hospital and Zhongshan Ophthalmic Center (2017KYPJ104).

DR patients with both type 1 diabetes and type 2 diabetes were included in this study. Diagnoses with diabetes were established according to the World Health Organization diagnostic criteria. Regular fundus photographs were taken from healthy people during their annual physical examinations. Exclusion criteria included: the refractive media were too cloudy to take a clear photograph; the diopter was greater than 6D; patients with systemic diseases other than diabetes that could also lead to ocular complications; patients with familial or hereditary ocular diseases; a history of ocular trauma; a history of medications that may cause ocular side effects (e.g., chloroquine, hydroxychloroquine, chlorpromazine, and rifampicin).

Before fundus photographing, participants would undertake slit-lamp and non-contact tonometer examinations. Tropicamide phenylephrine eye drops were applied for pupil dilation. When the pupil was dilated to be large enough (usually 8 × 8 mm^2^), a color fundus photograph would be taken for the participant using a fundus camera (Topcon, TRC-50DX, Japan). Images were saved in the JPG format (24-bit RGB), with a resolution of 2880 × 2136 pixels. Single-field central posterior 50° images, covering OD and macula, were analyzed in this study.

During the image quality control stage, we excluded 15 fundus images that are too blurry or of extremely large-area lesions (from the original selection). After image quality control, our dataset consists of 588 fundus images from DR patients and 631 fundus images from healthy people.

### Image categorization

**Fig. 2:**
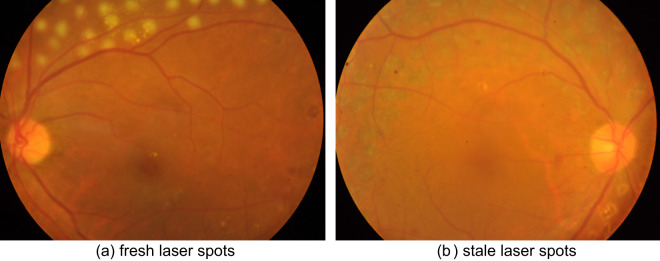
Fundus images of patients who were treated with retinal photocoagulation. After retinal photocoagulation, the lesions in the two images are relatively mild and can be classified as moderate non-proliferative DR (stage 2) even though the two patients should belong to stage 3 or stage 4 originally.

**Fig. 3:**
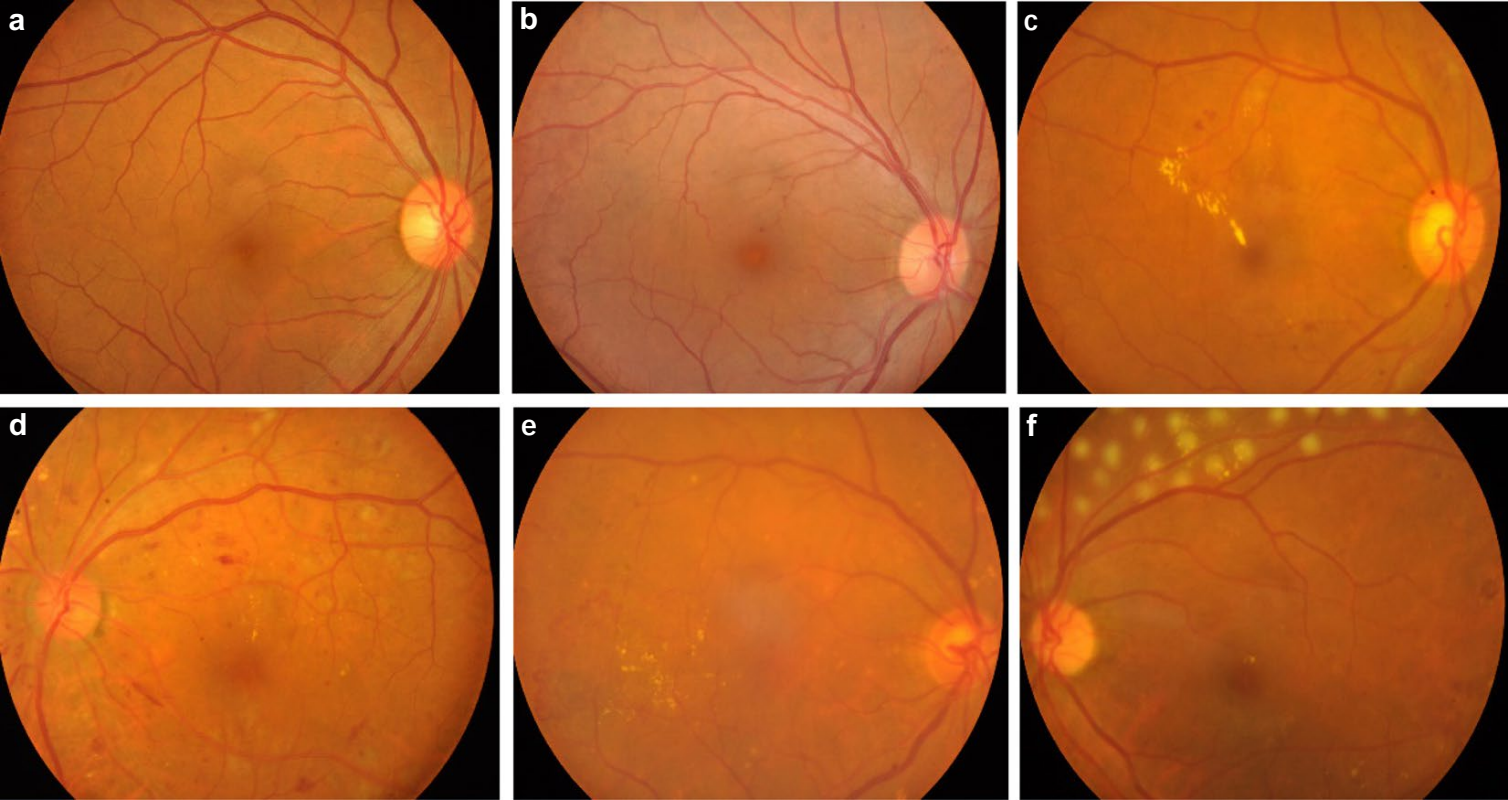
Color fundus images at different DR stages. (**a**) normal healthy fundus; (**b**) DR stage 1, mild non-proliferative DR, microaneurysms can be seen in the center; (**c**) DR stage 2, moderate non-proliferative DR, there are hard exudates in the center, several microaneurysms and patchy hemorrhage; (**d**) DR stage 3, severe non-proliferative DR, microaneurysms, hard exudates, cotton wool spots and patchy hemorrhages can be seen; (**e**) DR stage 4, proliferative DR, neovascularization can be seen in the inferotemporal quadrant; (**f**) this patient was treated with retinal photocoagulation, and fresh whitish laser spots can be seen on the superior retina.

The grading of DR refers to the International Clinical DR Severity Scale^31^. The only difference was that we considered healthy fundus photographs without diabetes as stage 0 instead of “diabetes patients with no apparent retinopathy”. And considering that some patients may have been treated with retinal photocoagulation and that laser spots or scars may affect staging and detection, we grouped fundus photographs with laser spots or scars into a separate category. Typically, the presence of laser spots or scars on a fundus image indicates that the patient is of severe non-proliferative DR or proliferative DR (stage 3 or stage 4). Some lesions may disappear after receiving retinal photocoagulation, and thus the grade determined from the fundus image may be inconsistent with the patient’s actual DR severity grade, such as samples shown in Fig. 2. Three experienced ophthalmologists at Zhongshan Ophthalmic Centre of Sun Yat-sen University performed screening and grading of the fundus photographs. Specifically, every fundus photograph was read by two ophthalmologists independently, then a third ophthalmologist would re-annotate the ones with inconsistent annotations from the previous two ophthalmologists. The entire dataset was distributed as follows: 631 photographs were confirmed as normal healthy fundus; 24, 365, 73 and 58 photographs were respectively classified to be mild non-proliferative DR, moderate non-proliferative DR, severe non-proliferative DR, and proliferative DR; and 68 photographs were classified to be DR with laser spots or scars (Table 1). Representative examples in each categorization are shown in Fig. 3. Additionally, we also provided DR grading labels for each fundus image according to the protocol from the American Academy of Ophthalmology and the Scottish DR grading protocol to facilitate comparisons of our dataset with other existing datasets^32–35^. Also, we provided DR grading labels for images in category 5 (fundus images with laser spots or scars) assessed by the three aforementioned protocols.

**Table 1:** Criteria of DR grading and the number of fundus photographs belonging to each category.

Classification/DR Grading	Findings Observable in Photographs	Number
0. normal healthy fundus	Without any lesions	631
1. mild non-proliferative DR	Microaneurysms only	24
2. moderate non-proliferative DR	More symptoms than just microaneurysms but less than severe non-proliferative DR	365
3. severe non-proliferative DR	One or more of the following:More than 20 intraretinal hemorrhages in each of 4 quadrants;	73
	- Definite venous beading in more than 2 q-uadrants;	
	- Prominent intraretinal microvascular abn-ormalities in more than 1 quadrant and no signs of proliferative DR	
4. proliferative DR	One or more of the following:	58
	- Neovascularization;	
	- Vitreous/preretinal hemorrhage	
5. DR with laser spots/scars	DR accompany with whitish laser spots or grey laser scars	68

**Fig. 4:**
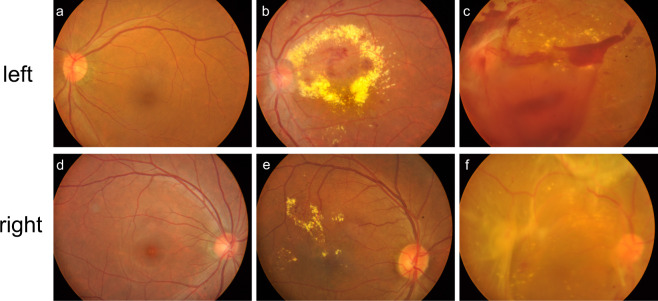
Representative fundus images from left eyes and right eyes. Examples include normal fundus photographs (**a**,**d**), clear fundus photographs with DR (**b**,**e**), and blurry fundus photographs due to proliferative DR (**c**,**f**).

Distinguishing whether a fundus image comes from a left eye or a right eye is one of the first steps in ophthalmic examinations. Generally, for most fundus images in the categories of stage 0 to stage 3, the left eye and right eye can be easily distinguished according to OD’s position and the direction of the retinal vessels, although there may be lesions existing. As shown in Fig. 4, in some cases of proliferative DR, the fundus images become blurry due to large-scale hemorrhages and exudates, and ODs become less prominent. In those cases, the left and right eyes can still be distinguished based on the residual blood vessel traces. Table 2 tabulates the numbers of left eye and right eye fundus images in each of the 5 stage categories as well as category 5 (DR with laser spots or scars). Overall, in terms of left-versus-right eye classification, our dataset is relatively balanced.

**Table 2:** he numbers of left eye and right eye fundus images within each of the 6 categories (stage 0 to stage 4 and category 5).

Category	Stage 0	Stage 1	Stage 2	Stage 3	Stage 4	Category 5	Total
Left eyes	323	10	193	37	12	32	607
Right eyes	308	14	189	41	31	29	612

### Creation of annotations for exudate detection

**Fig. 5:**
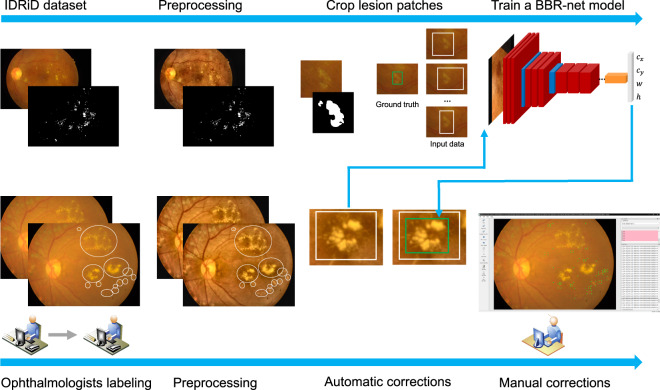
The flowchart of generating the ground truth bounding boxes of exudates.

**Fig. 6:**
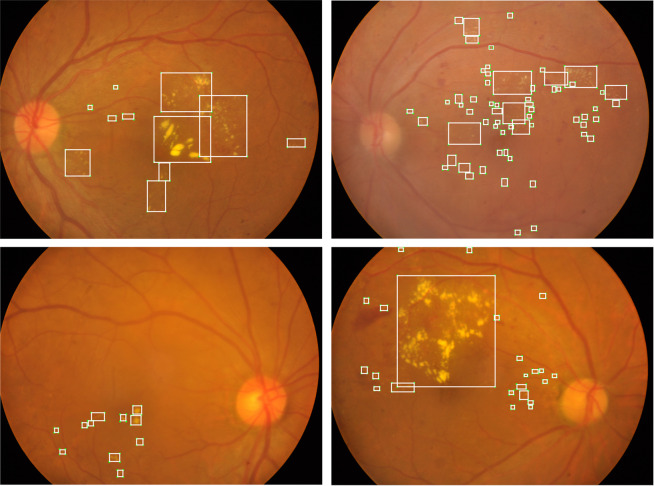
Representative examples of exudate detection labels.

**Fig. 7:**
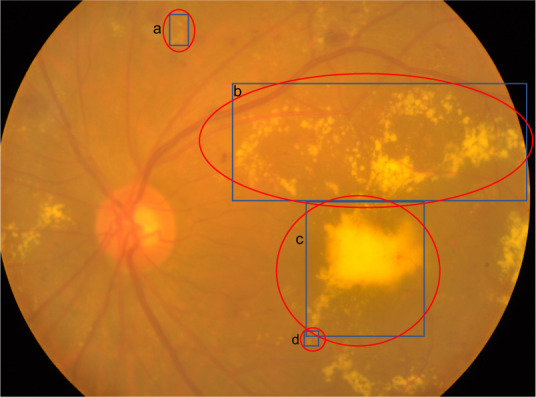
Several examples to help illustrate the labeling criteria for exudates.

As mentioned in the above subsection, fundus photographs of stage 0 are normal healthy fundus with no lesions and stage 1 fundus images contain only microaneurysms. Therefore, we only prepared the ground truth detection bounding boxes for exudates (including hard exudates and cotton wool spots) in fundus images of stage 2, stage 3, stage 4, as well as those with laser spots or scars, ending up with a total of 564 fundus images. In this work, we labeled the exudates according to the most common format in computer vision detection tasks, namely bounding boxes. As shown in Fig. 5, the entire annotation procedure went through the following four steps: (1) An experienced ophthalmologist from Zhongshan Ophthalmic Centre screened fundus images with exudates and identified them in the form of a coarse bounding circle, and then another ophthalmologist inspected the bounding circle and corrected if necessary, such as missing labels and incorrect labels; (2) Images identified to have exudates labels went through contrast limited adaptive histogram equalization (CLAHE) and adaptive gamma correction with weighting distribution (AGCWD) as preprocessing for the purpose of contrast enhancing and illumination correction^36,37^; (3) A bounding box refining network (BBR-net) model (trained from the IDRiD dataset^28^) was employed to refine coarse bounding boxes (generated from coarse bounding circles in step (1) into more precise bounding boxes (the four sides of the refined boxes were much closer to the boundary of each lesion area than the coarse ones); (4) A third ophthalmologist re-checked the output of the aforementioned model and made manual corrections again. Detailed information of step 2 and step 3 can be found in our previous work^38^. Representative examples of exudate detection labels are shown in Fig. 6. All clinicians involved in exudate labeling followed the following criteria:

- For relatively independent but still connected lesions, regardless of size and shape, in step (1), the boundary circle should include the entire area of the lesion. In step (4), the bounding box should be as close as possible to the edge of each exudate.
- For a large and coarsely-connected lesion, there may be multiple smaller lesions inside. However, if the smaller sub-lesions are very close to each other and it is challenging to identify every single sub-lesion, they can be grouped and considered as one single lesion, as shown in exudates a and b in Fig. 7.
- If the lesion label obtained from the above criterion 2 is very large such that there are a lot of background pixels included, the ophthalmologists separate it to be two exudate labels according to an appropriate boundary separation rule, as exudates b and c in Fig. 7 show.
- Overlap between two exudate labels is allowed, such as exudates c and d in Fig. 7 show. The ophthalmologists only need to make sure that the boundary circle completely contains the exudate, and the bounding box contours the boundary of each exudate as close as possible.
- In terms of other special cases, the ophthalmologists communicate with each other to reach consistent labeling criteria.

### Creation of OD bounding box and fovea location annotations

**Fig. 8:**
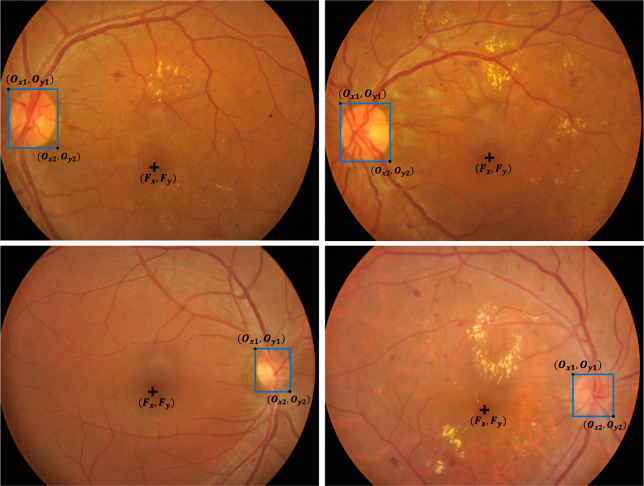
Representative instances of the OD and fovea annotations.

Along with the annotations presented above, this dataset also provided center pixel locations of fovea (*F*_*x*_,*F*_*y*_) as well as bounding boxes of ODs (*O*_*x*1_, *O*_*y*1_, *O*_*x*2_, *O*_*y*2_) for all images. The procedure of creating those two labels consisted of the following two steps: automatic generation and manual correction. OD and fovea are two of the most important anatomical landmarks of fundus images. In one of our previous works^39^, we trained a region proposal network and a cascaded network for automated OD detection in the form of a bounding box and fovea localization in the form of a pixel location identification. After that, an ophthalmologist visually examined the accuracy of the automatic results and performed manual corrections if necessary. The OD bounding box should be the smallest rectangle that bounds the OD and the fovea is defined to be the center of the macula. Figure 8 shows representative instances of the OD and fovea annotations.

## Data Records

This dataset is publicly available at https://www.aiforeye.cn/ and 10.6084/m9.figshare.12570770.v1^40^, which is stored as a zip file. In the unzipped folder, all the raw fundus images, the exudate annotations, the DR grading labels, and the OD and fovea location annotations are stored in three subfolders, namely “originalImages”, “exudateLabels”, and “odFoveaLabels”. In the “originalImages” folder, files are saved in the JPG format and named as “*n*.jpg”, with *n* ranging between 0001 and 1219 indicating the *n*^*th*^ sample. In that folder, we also provide a comma-separated-values (CSV) file named “drLabels.csv”, wherein the first column indicates the file name, the second column indicates the left-versus-right eye categories with 0 representing left eyes and 1 right eyes, the third column indicates the DR category assessed via the International Clinical DR Severity Scale (0 to 5, with 0 representing normal healthy, and 1 to 5 respectively representing mild non-proliferative DR, moderate non-proliferative DR, severe non-proliferative DR, proliferative DR, and DR with laser spots or scars), the fourth column indicates the DR grade assessed via the American Academy of Ophthalmology protocol, and the fifth column indicates the DR grade assessed via the Scottish DR grading protocol. Another CSV file named “c5_DR_reclassified.csv” provides the DR labels for images belonging to category 5 assessed via the three aforementioned protocols. The exudate detection labels, OD bounding box’s coordinates, as well as fovea location’s coordinates are saved in the XML format stored at the corresponding folders (namely “exudateLabels” and “odFoveaLabels”), following the same specifications as the Pascal Voc dataset^41^. Hard and soft exudates are labeled separately in this dataset. In the XML files, “ex” stands for hard exudates and “se” for soft exudates.

## Technical Validation

It is worth mentioning that although some degree of automation was involved in generating all four types of labels provided in this work, expert verification was always performed as the last step to ensure the quality and correctness of the annotations.

For the OD bounding box and fovea location labels, they are relatively simple and had been labeled in a semi-automated manner. Specifically, automated OD bounding boxes and fovea locations were obtained from a deep learning model^39^, the performance of which had been verified on a large set of fundus images. After that, one ophthalmologist checked the results and corrected if necessary. For the left-versus-right eye label, the definition is very straightforward, according to OD’s position and direction of the retinal vessels. Every fundus photograph was independently read by two ophthalmologists, and then a third one would re-annotate the ones with inconsistent judgments. For this label, the value of intra-class correlation coefficient (ICC)^42^ between the initial two ophthalmologists is 1 and thus the third ophthalmologist was not involved at all. For the DR grade label, the ICC between the initial two annotators is 0.91. The main difficulty lies in distinguishing between mild non-proliferative DR, moderate non-proliferative DR, and severe non-proliferative DR. For the exudate annotation, we calculate the Dice coefficient^43^ between two exudate labels (boundary circle labels are transformed into binary masks, where the pixel value inside the circle is 1 and the pixel value outside the circle is 0) to assess the inter-rater agreement, and the mean Dice value between the initial two annotators is 0.89. In conclusion, for the four kinds of labels provided in our dataset, different annotators had high consistency/inter-rater agreement, ensuring the high quality of the annotations of our proposed SUSTech-SYSU dataset.

When constructing the exudate annotations, we also trained a BBR-Net model based on the exudate labels provided in the IDRiD dataset (combining soft exudates and hard exudates together). Evaluated on the IDRiD dataset, our BBR-Net can effectively refine coarse exudate annotations, with the average intersection-over-union (IoU)^44^ being 0.8653 when compared with well-annotated bounding boxes (generated from the pixel-wise labels provided in IDRiD). Then, we applied the trained and validated BBR-Net to the automatic correction step in exudate label creation in this work. Additionally, experienced ophthalmologists have visually examined the quality of all 1219 fundus images used in this study to ensure adequate image quality. Our *aiforeye* platform also embedded a function of automated quality assessment for fundus images.

In order to quantify the relationship between lesion area and DR grade in the provided dataset, we calculate the total number, average number, total area, and average area of exudates contained in images belonging to each category of the provided dataset, which are demonstrated in Tables 3 and 4. Our entire dataset contains 15,652 exudates, and the total number of pixels inside all exudate bounding boxes are 212201128, accounting for 6.11% of the total area (3469547520). All these metrics were computed from the 564 fundus images with exudate annotations. It can be easily seen that the data in those two tables are in line with clinical knowledge. Many fundus images in the category of stage 4 had severe fibrous proliferation or severe vitreous hemorrhage, which obscured exudates. Therefore, the average area of exudates is the largest for images in the category of stage 3. The average area of either stage 2 or stage 4 is less than that of stage 3. After receiving retinal photocoagulation treatment, the number of exudates decreased and the average area is smaller than both stage 3 and stage 4.

**Table 3:** The total number of exudates contained in fundus images belonging to each category of this dataset.

Category	Stage 2	Stage 3	Stage 4	Category 5	Total
Total	9,156	2,847	1,532	2,117	15,652
Average	25.08	39.00	26.41	31.13	27.75

**Table 4:** The area (pixel numbers) of exudates contained in fundus images belonging to each category of this dataset.

Category	Stage 2	Stage 3	Stage 4	Category 5	Total
Total	123,470,897	40,280,698	22,968,498	25,481,035	212,201,128
Average/lesion	13,485.24	14,148.47	14,992.49	12,036.39	13,557.44
Average/image	338,276.43	551,790.38	396,008.59	374,721.10	376,243.13

Even though the exudate detection labels were generated under the unanimous determination of three ophthalmologists, for exudates the edges of which are often not distinct or cover a large area, it is sometimes difficult to determine and justify which pixels should be included in a single bounding box. Therefore, there is still a certain degree of subjectivity in our exudate annotations. Finding a proper balance between pixel-level segmentation labeling and bounding box detection labeling is one of our future research directions.

Although our provided dataset is quality controlled, individual fundus images are relatively variable in terms of quality. Some are blurrier than others. With that being said, providing a large dataset containing both high-quality and relatively low-quality samples ensures more realistic model training so as to accommodate real clinical scenarios. In addition, this dataset may be also useful for advancing automated quality-enhancement techniques^45,46^ for fundus images, especially in the context of DR screening.

Compared with the 81 samples in the IDRiD dataset and the 47 samples in the e-Ophtha EX dataset, the dataset we introduced in this paper has a relatively large sample size (564 samples in total) in terms of exudate detection tasks. However, in terms of DR classification and grading, this dataset is unbalanced to a certain extent, and the sample sizes of specific categories are relatively limited (mild non-proliferative DR, severe non-proliferative DR, and proliferative DR). In this case, training with machine learning, especially deep learning methodologies, may cause over-fitting problems. As such, in terms of the development of automated DR classification algorithms, this dataset may be more suitable for applying “Few-shot Learning” methods, the research topic of which has gradually received extensive attention and developments in the past few years^47^. One of our future research efforts is to address this limitation.

## Usage Notes

This dataset can be downloaded through the link mentioned above. Users of this dataset are expected to cite this paper in any research output generated from using this dataset as well as appropriately acknowledge the contributions of this dataset.

After copying all images from the “originalImages” folder to the “exudateLabels” and “odFoveaLabels” folders, users can directly open the provided fundus images and the corresponding exudate detection labels, OD bounding box’s coordinates, as well as fovea location’s coordinates using *Labelimg*^48^ (a graphical image annotation tool, which can be accessed at https://github.com/tzutalin/labelImg). This tool provides functions of visualizations and modifications of annotations (according to research needs). Please note in order to display directly in *Labelimg*, the fovea location’s coordinates are transformed into a small box (*F*_*x*_, *F*_*y*_, *F*_*x*+1_, *F*_*y*+1_).

## Data Availability

In the process of constructing the dataset provided in this work, we used several automatic algorithms developed in our previous works^38,39^. The source code for the bounding box refining network (BBR-net) can be accessed at https://github.com/YijinHuang/BBR-Net (or 10.5281/zenodo.4041331)^49^ and code for OD detection and fovea localization are available upon request. Also, we have embedded all involved algorithms into a cloud platform that we developed. Users of this dataset can access the two automatic algorithms by visiting our website at https://www.aiforeye.cn/ and uploading fundus images for analysis. The functions provided by our platform include classification of left and right eyes, DR grading, lesion detection, identification of OD and fovea, as well as some additional functions such as retinal vessel segmentation and statistical analyses of vessel morphometrics and lesion abnormalities. Please note that our algorithms for segmenting and classifying corneal ulcers from ocular staining images (the dataset we published before)^21^ can also be accessed on this platform.
